# Clinical accuracy of transferring virtually planned zygomatic implant positions in severely atrophic maxilla: a single-arm study

**DOI:** 10.1186/s12903-026-10079-x

**Published:** 2026-11-03

**Authors:** Mahmoud Mohamed Hassan Agha, Ahmed Abdelmohsen Younis Ahmed Ali, Mohamed Ahmed ElSholkamy, Amr Aly El Swify

**Affiliations:** 1 Faculty of Dentistry, Suez Canal University, Ismailia, Egypt; 2 Oral and Maxillofacial Surgery Department, Faculty of Dentistry, Suez Canal University, Ismailia, Egypt

**Keywords:** Zygomatic implants, Virtual planning, Atrophic maxilla, Cone-beam computed tomography, Computer-guided surgery, Implant accuracy

## Abstract

**Objectives:**

This study aimed to evaluate the clinical accuracy of transferring virtually planned zygomatic implant positions in severely atrophic maxilla.

**Materials and methods:**

This clinical accuracy study included 24 participants with severely atrophic edentulous maxilla. All cases were digitally planned using cone-beam computed tomography (CBCT) and treated with bilateral zygomatic implants under general anesthesia, with additional anterior conventional implants. Postoperative CBCT was used to superimpose the actual implant positions onto the virtual plan and assess coronal and apical deviations.

**Results:**

A total of 48 zygomatic implants were placed in 24 participants. Three-dimensional superimposition of the preoperative virtual plans and postoperative CBCT scans demonstrated a mean coronal deviation of 1.917 ± 0.401 mm overall, with values of 1.989 ± 0.540 mm on the right side and 1.846 ± 0.160 mm on the left side. The overall mean apical deviation was 1.733 ± 0.429 mm, with values of 1.757 ± 0.172 mm on the right side and 1.709 ± 0.588 mm on the left side.

**Conclusions:**

Virtual surgical planning enabled the transfer of planned zygomatic implant positions with measurable coronal and apical deviations. These findings describe short-term positional accuracy and should not be interpreted as evidence of long-term implant stability, treatment efficacy, or overall safety.

**Trial registration:**

Trial was retrospectively registered on 31/03/2023 at Pan African Clinical Trials Registry (https://pactr.samrc.ac.za) (ID: PACTR202603707948061) under the following link: https://pactr.samrc.ac.za/TrialDisplay.aspx?TrialID=40945.

## Introduction

Dental implants have transformed oral rehabilitation since the landmark work of Brånemark in the late 1970s, establishing endosseous titanium fixtures as the cornerstone for restoring function and aesthetics in edentulous and partially edentulous patients [1]. Long-term data demonstrate high survival rates at both implant and patient levels; however, therapeutic success is contingent upon a multifactorial interplay of biological and mechanical determinants, including systemic health, bone quantity and quality, surgical protocol, prosthetic design, and patient-related behavioral factors such as oral hygiene compliance. Inadequate plaque control remains a leading contributor to early implant failure, emphasizing the need for comprehensive preoperative risk assessment and individualized treatment planning [2]. 

Rehabilitation of the severely atrophic maxilla represents one of the most demanding challenges in implant dentistry, where post-extraction alveolar resorption imposes substantial constraints on conventional implant placement [3]. Physiologically, the extraction socket undergoes differential healing, with osseous fill predominantly in the apical two-thirds and fibrous connective tissue occupancy in the coronal portion [4]. Longitudinal investigations by Tallgren revealed that alveolar bone loss during the first year after tooth extraction is approximately tenfold greater than in subsequent years, with the anterior maxilla exhibiting up to 40% reduction in vertical height and 60% reduction in horizontal width within the initial 6 months [5]. Concurrently, progressive pneumatization of the maxillary sinus further diminishes available bone height in the posterior maxilla, frequently yielding insufficient vertical and horizontal dimensions for standard implant placement without adjunctive augmentation [6]. 

Conventional management of severe maxillary atrophy has relied on bone augmentation strategies, including maxillary sinus floor elevation, onlay grafting using autogenous bone from intraoral or extraoral donor sites, and guided bone regeneration [7]. Although these approaches provide viable reconstructive options, they are associated with notable limitations that may compromise patient acceptance and treatment efficiency [8]. The requirement for multiple surgical stages, donor-site morbidity particularly with extraoral harvest sites such as the iliac crest and prolonged healing intervals for graft maturation collectively extend treatment duration, increase costs, and elevate patient burden [9]. These constraints have motivated the development of alternative therapeutic paradigms that circumvent the need for extensive grafting while maintaining predictable clinical outcomes [10]. 

The concept of (remote implant support) heralded a paradigm shift in the management of severe maxillary atrophy, most prominently through the introduction of zygomatic implants by Brånemark and colleagues [11]. Zygomatic implants are specialized extra-long fixtures, typically ranging from 30 to 52 mm in length, with diameters of 4–5 mm and a coronal head angulated at approximately 45° with an external hex connection [12]. These implants are designed to engage the zygomatic bone as an alternative anchorage site, with the implant head positioned at the alveolar crest in the posterior maxilla and the apex secured within the dense cortical bone of the zygoma [13]. This anatomical trajectory bypasses the deficient alveolar ridge and maxillary sinus, offering a graftless solution for patients with minimal residual bone [14]. 

Current indications for zygomatic implants include cases in which both bone height and width are reduced to ≤ 4 mm, precluding conventional implant placement without prior augmentation [15]. Despite the relatively trabecular character of zygomatic bone, which is generally considered less favorable for osseointegration, zygomatic implants achieve survival rates approaching 97% [16]. This favorable outcome is attributed to their unique biomechanical engagement of four distinct cortical bone plates along their trajectory from the palate to the zygoma, conferring superior primary stability [17]. Consequently, zygomatic implant therapy eliminates the need for sinus augmentation and bone grafting procedures, substantially reducing surgical morbidity, treatment time, and overall costs, while enabling immediate or early loading protocols in selected cases [18]. Recent clinical series have described favorable 3‑ to 5‑year radiologic and clinical outcomes for immediate full‑arch restorations supported by conventional implants combined with unilateral or bilateral zygomatic implants, supporting the longer‑term predictability of such hybrid graftless protocols [19]. 

Nonetheless, the surgical placement of zygomatic implants entails distinct technical challenges and potential complications that demand meticulous execution [20]. The implant trajectory traverses near critical anatomical structures, including the infraorbital neurovascular bundle, the maxillary sinus, and the orbital cavity [21]. Reported complications include oro-antral communication, infraorbital nerve paresthesia or altered sensation, peri-implant soft tissue disturbances, and, rarely, orbital penetration [22]. Among these, maxillary sinusitis represents the most frequently documented complication, with incidence rates reported up to 37.5% in some series [23]. The considerable length of these implants and the complexity of their three-dimensional positioning underscore the technique-sensitive nature of the procedure and the potential for clinically significant deviation from the planned trajectory [24]. 

In response to these challenges, the integration of digital technologies and minimally invasive surgical principles has transformed zygomatic implant therapy [25]. Virtual surgical planning based on cone-beam computed tomography (CBCT) data enables precise three-dimensional assessment of maxillofacial anatomy and accurate preoperative determination of optimal implant position and length [26]. Computer-guided surgery facilitates prosthetically driven implant placement while minimizing the risk of injury to adjacent vital structures [27]. The rapid advancement of static surgical guides and navigation systems has established computer-assisted implantology as an emerging standard of care, offering enhanced predictability, reduced operative time, and decreased postoperative morbidity [28]. Given these technological advances, the present study aims to evaluate the clinical accuracy of transferring virtually planned zygomatic implant positions in severely atrophic maxilla. The null hypothesis is that there is no significant difference between the virtually planned and the clinically achieved three-dimensional positions of zygomatic implants in terms of linear (entry point, apex).

## Materials and methods

### Sample size and recruitment

**Fig. 1:**
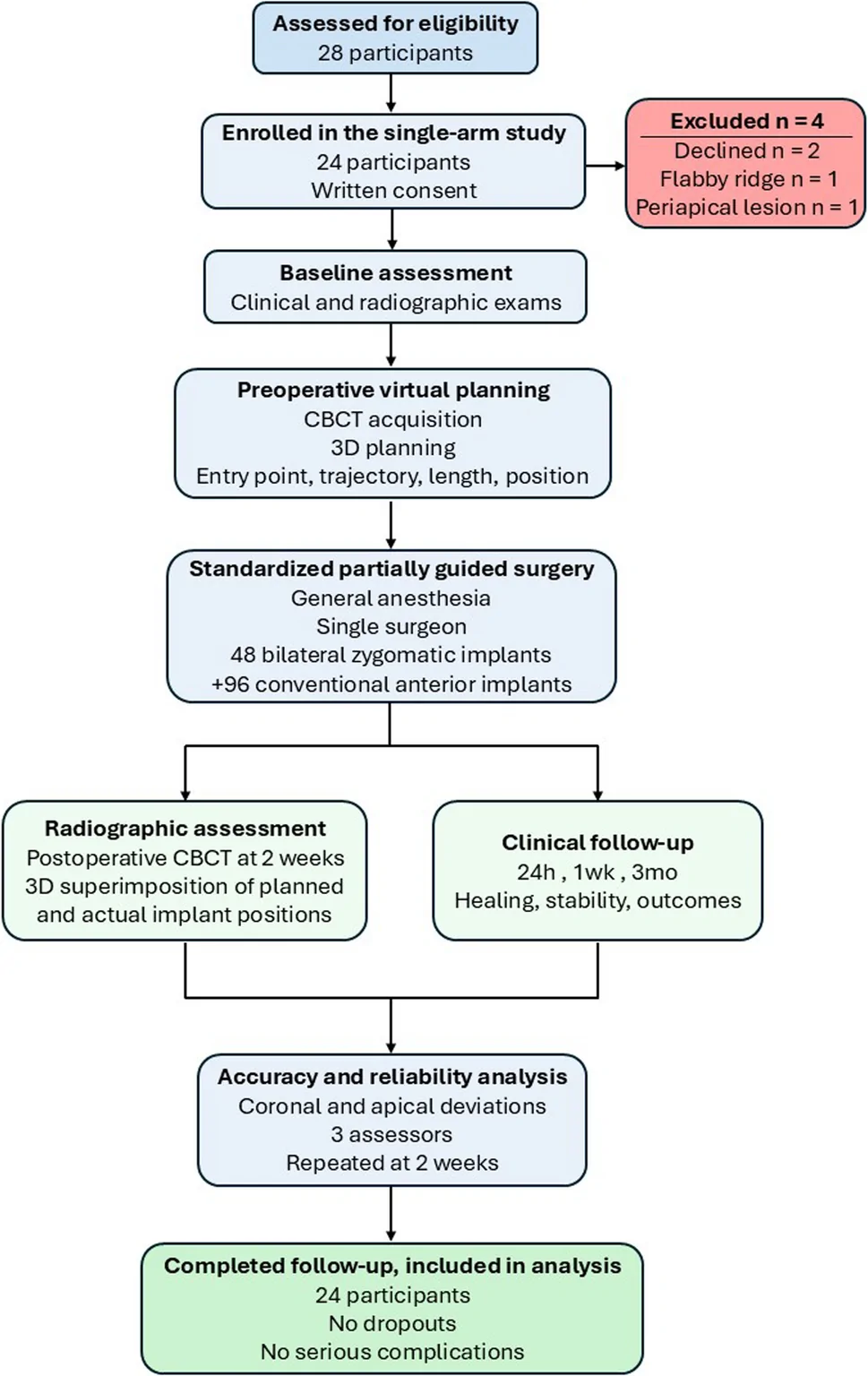
Participant flow and study procedures

Sample size calculation, performed using G*Power software version 3.1.9.7, determined that a minimum cohort of twenty-four participants would provide adequate statistical power (1 − β = 0.80) to detect an effect size of 0.60 at a two-sided significance level of α = 0.05, assuming a standard deviation of 1.3; a 20% dropout adjustment was incorporated, Fig. 1 [29].

### Participant involvement and eligibility criteria

Eligible participants were adults aged 18 years or older, medically fit for surgery under general anaesthesia, with severely atrophic edentulous maxilla as residual posterior maxillary alveolar bone height and width of ≤ 4 mm at the intended conventional implant sites, as measured on preoperative CBCT images, that could not be restored through conventional treatment modalities, and who demonstrated good compliance and oral hygiene habits. Exclusion criteria encompassed acute sinusitis, intraoral pathological lesions related to the maxillary sinus or zygoma, significant systemic medical compromise, heavy smoking exceeding 2 packs per day, restricted mouth opening with an interincisal distance of less than 3 cm, and an interarch distance below 22 mm. Of twenty-eight initially assessed participants, two declined participation and two were subsequently excluded, one due to a flabby ridge that precluded accurate soft tissue support for the surgical guide, and another due to a large periapical lesion in the left maxillary premolar region.

### Trial design, setting, and randomization

This surgical accuracy study was carried out in the Department of Oral and Maxillofacial Surgery, Faculty of Dentistry, Suez Canal University, Egypt. Recruitment commenced on (06/05/2023) and was completed on (14/08/2025). Therefore, registration occurred after the enrollment of the first participant and is accurately described as retrospective registration. All participants followed an identical treatment protocol comprising preoperative virtual implant planning, surgical placement of zygomatic implants, and postoperative radiographic evaluation. Because the study employed a single-arm accuracy design, no randomization into separate intervention groups was undertaken. Instead, each participant functioned as their own control, with the virtually planned implant positions directly compared to the actual postoperative implant positions to assess the accuracy of the virtual surgical planning [30].

### Intervention

All participants followed a uniform treatment protocol. Preoperative cone-beam computed tomography (CBCT) images were acquired and imported into Blue Sky Plan software (Version 5.0, 2026, Grayslake, IL: Blue Sky Bio) to perform three-dimensional (3D) virtual planning of zygomatic implant placement. Implant size, entry point, trajectory, and final position were determined in accordance with the Zygoma Anatomy-Guided Approach (ZAGA) principles. Each planned zygomatic implant site was assessed independently on the preoperative CBCT images by the operating surgeon and classified as ZAGA type 0, 1, 2, 3, or 4 according to the morphology and degree of concavity of the lateral maxillary wall and the corresponding planned implant trajectory. Because the right and left sides could have different anatomical configurations, ZAGA classification was recorded at the implant-site level, Table 2 [31].

A static computer-guided protocol was used. A mucosa-supported, stereolithographically fabricated surgical guide was designed in BlueSky Bio software using merged dual-scan CBCT datasets and 3D-printed from biocompatible (MED610; Stratasys Ltd., Eden Prairie, MN, USA), incorporating 4-mm metal sleeves with an incomplete extension for trajectory monitoring. Although an anchor pin was included, its use for guide fixation requires clarification. Zygomatic and maxillary osteotomies were prepared sequentially with 2.70-/2.90-mm drills under copious irrigation; conventional implant osteotomies used 2.30-/2.80-/3.50-mm drills with corresponding guide keys. Drilling was guided, but implants were manually inserted after guide removal, classifying the procedure as partially guided.

All surgical procedures were performed by a single experienced implant surgeon with more than 10 years of experience in guided implant surgery. The virtual implant planning was performed by the same surgeon using the planning software.

**Fig. 2:**
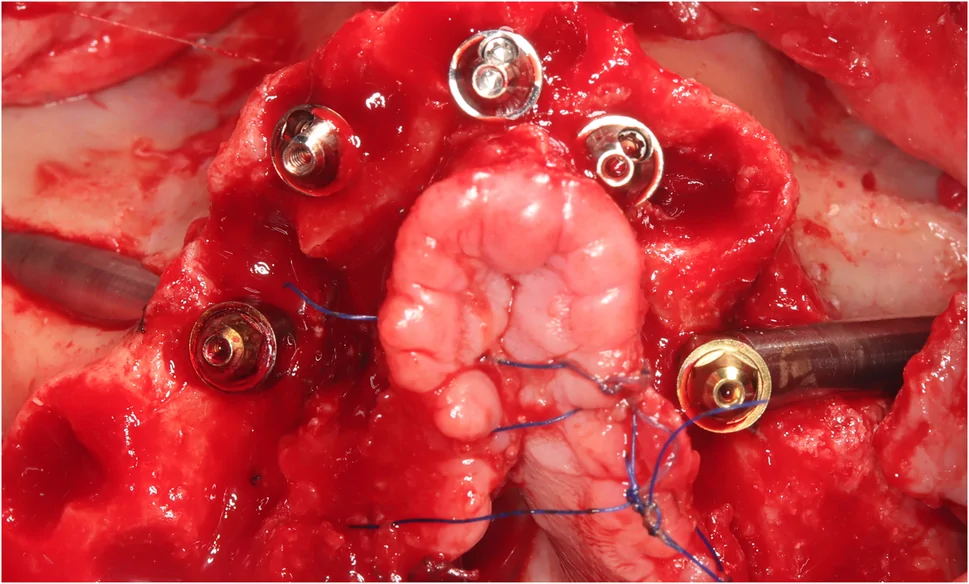
Intraoperative view of zygomatic implant placement

Surgery was carried out under general anesthesia using a standardized operative technique. After raising a mucoperiosteal flap and exposing the zygomatic bone, osteotomies were prepared following the manufacturer’s recommended drilling sequence, and zygomatic implants (JDZygoma, JDentalCare S.r.l., Modena, Italy) were inserted at the preplanned sites, Fig. 2. Where indicated, conventional implants were placed in the anterior maxilla to enhance prosthetic support. Following implant insertion, the surgical sites were irrigated, the buccal fat pad was mobilized to cover any exposed implant shafts when necessary, and the flaps were repositioned and sutured [32].

**Fig. 3:**
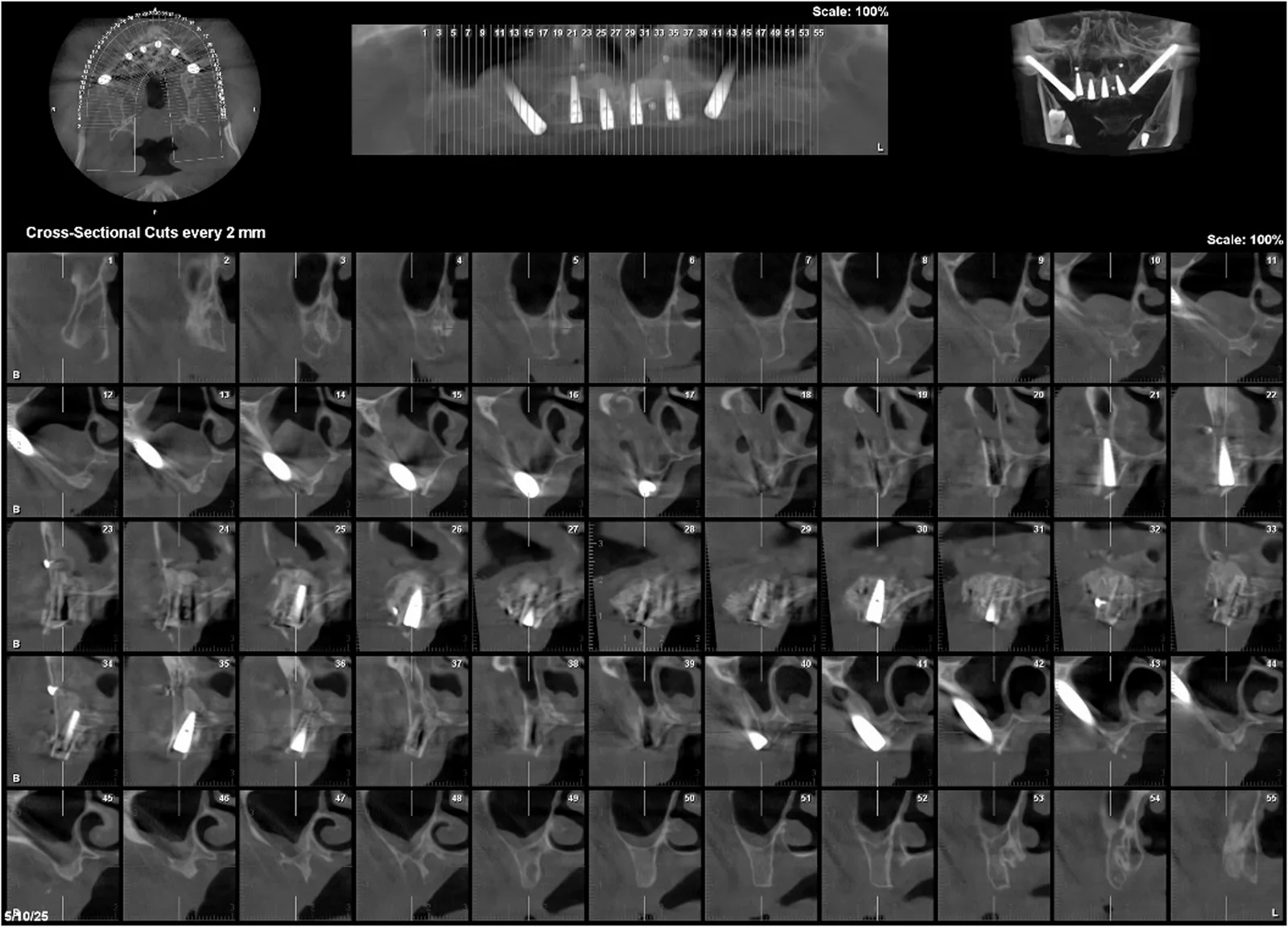
Postoperative CBCT radiographs following implant placement

In the postoperative period, all participants received an identical medication regimen and follow-up schedule. A postoperative CBCT scan was obtained two weeks after surgery, Fig. 3. The actual implant positions were then superimposed onto the preoperative virtual plan using Mimics Medical and 3-matic Medical software to evaluate planning accuracy. The primary accuracy metrics assessed were coronal deviation and apical deviation between the planned and actual implant positions [33].

### Prosthetic rehabilitation and loading protocol

Following implant placement, the participants received a provisional PMMA screw-retained, full-arch maxillary prosthesis supported by 2 zygomatic implants and 4 conventional anterior implants through multi-unit abutments. The prosthesis was delivered within 24 h, following an immediate loading protocol. Participants were instructed to maintain a soft diet for the first 8 weeks. Prosthetic rehabilitation was considered successful when the prosthesis was delivered and remained clinically stable and functional without implant mobility, prosthesis instability, pain, infection, or mechanical complications.

### Participant timeline

Following ethical approval and the acquisition of written informed consent, baseline clinical and radiographic assessments were performed. Virtual planning of zygomatic implant placement was then carried out. Subsequently, all participants underwent standardized surgical placement of zygomatic implants under general anesthesia. Preoperative and postoperative CBCT examinations were performed using a (Viso G7; Planmeca). The scans were acquired using a (field of view: 15 mm × 13 mm), isotropic voxel size of 0.3 mm³, tube voltage of 105.0 kVp peak, and tube current of (8.0 mA). The exposure time was (12 s). Images were reconstructed using (3D imaging software; Romexis), with a slice thickness and reconstruction interval of (0.1 mm and 2 mm, respectively). The patient’s head was positioned according to the manufacturer’s standard protocol, and the images were obtained with the patient in a stable and reproducible position. It was obtained 2 weeks after surgery to assess implant placement accuracy by comparing the actual implant positions with the preoperative virtual plan. Clinical follow-up visits were scheduled at 24 h, 1 week, and 3 months postoperatively to evaluate healing, complications, implant stability, and prosthetic outcomes [34].

### Outcomes, data collection methods, and management

Accuracy was quantified using 3D measurements of coronal deviation and apical deviation [35]. Baseline demographic and clinical information were recorded at enrollment, in Table 1. The postoperative CBCT datasets were aligned with the preoperative virtual planning models using Mimics Medical 19.0 and 3-matic Medical 14.0 software. Accurate registration was achieved using fixed anatomical landmarks, and standardized reference planes (mid-sagittal, Frankfort horizontal, and coronal planes) were established to enable consistent 3D measurements. Linear distances from the implant platform and apex to the reference planes, as well as direct linear between the planned and actual implant positions, were measured and recorded [36].

**Table 1:** Participant demographics and implant characteristics

Characteristic	Value
Total participants, *n*	24
Male / Female, *n*	12 / 12
Age (years), mean ± SD (range)	41.9 ± 14.9 (20–61)
Zygomatic implants placed, *n*	48
Zygomatic implant length (mm), mean ± SD (range)	41.7 ± 2.9 (37.5–47.5)
Zygomatic implant diameter (mm)	4.0
Conventional implants placed, *n*	96
Conventional implant length (mm), mean ± SD (range)	9.9 ± 1.4 (8.5–12.0)
Conventional implant diameter (mm)	3.8

All radiographic assessments were performed and clinical follow-up evaluations were conducted to monitor healing, swelling, pain, edema, bleeding, neurosensory disturbances, implant stability, and prosthetic outcomes [37].

### Assessment of implant deviations

The planned and clinically achieved implant positions were then superimposed and evaluated using the same three-dimensional coordinate system and reference planes. The coronal reference point was defined at the implant platform or implant entry point, whereas the apical reference point was defined at the terminal apical end of the implant [37].

Coronal and apical deviations were calculated as absolute linear distances between the corresponding planned and actual reference points. Accordingly, these measurements represented the magnitude of positional discrepancy and were not signed coordinate differences or three-dimensional Euclidean distances. The coronal deviation was measured in the horizontal reference plane at the implant entry point, and the apical deviation was measured in the horizontal reference plane at the implant apex. The corresponding formulas were: 𝐷 coronal = ∣ 𝐶 actual – 𝐶 planned ∣, 𝐷 apical = ∣ 𝐴 actual – 𝐴 planned ∣, where 𝐶 represents the coronal reference point and 𝐴 represents the apical reference point [31].

Thus, coronal and apical deviations were reported in millimetres. All measurements were obtained using the measurement tools of the planning and implant-analysis software after image superimposition.

Radiographic measurements were independently performed by 3 calibrated experienced assessors in 3D implant-position analysis. Assessors were blinded to the planned positional measurements and calibration was conducted using 20% randomly selected implants prior to the definitive assessment. To evaluate measurement repeatability, the selected implants were remeasured after 2 weeks under identical conditions, without access to the initial measurements.

### Statistical methods

Statistical analyses were conducted using JASP (JASP Version 0.98.1, JASP Team 2026). The dataset was checked for completeness, consistency, and potential data-entry errors before analysis. Continuous variables were summarized as mean ± standard deviation (SD), together with 95% confidence intervals (CIs), medians, interquartile ranges, and ranges where appropriate. Categorical variables were presented as frequencies and percentages. Distributional assumptions were assessed using histograms, Q–Q plots, and the Shapiro–Wilk test. Potential outliers were screened using boxplots and the 1.5 × interquartile-range rule. Valid extreme observations were retained and were not removed solely because of their magnitude [38].

Measurement reliability was assessed using intraclass correlation coefficients (ICCs). Intraobserver reliability was evaluated by comparing the baseline and two-week repeat measurements performed by each observer. Interobserver reliability was assessed among the three observers. A two-way, absolute-agreement ICC model was used, with 95% CIs reported for all ICC estimates. ICC values > 0.90 were interpreted as excellent reliability.

## Results

A total of 24 participants completed the study, comprising 12 males and 12 females. The mean age was 41.9 ± 14.9 years, with an age range of 20–61 years. Each participant received two zygomatic implants, resulting in a total of 48 zygomatic implants, in addition to 4 conventional anterior maxillary implants per participant, resulting in 96 conventional implants. The mean length of the zygomatic implants was 41.7 ± 2.9 mm, with a range of 37.5–47.5 mm, and all zygomatic implants had a diameter of 4.0 mm. The conventional implants had a mean length of 9.9 ± 1.4 mm, ranging from 8.5 to 12.0 mm, and a diameter of 3.8 mm (Table 1).

The ZAGA classification demonstrated that Type 3 was the most frequently observed anatomical pattern, accounting for 8 of 24 participants (33.3%), followed by Type 2 in 7 participants (29.2%). Type 1 and Type 4 were each observed in 4 participants (16.7%), whereas Type 0 was identified in 1 participant (4.2%) (Table 2).

**Table 2:** Participants’ ZAGA classifications

Characteristic	Category or assessment time	*n*/*N*	%
ZAGA classification	Type 0	1/24	4.2
	Type 1	4/24	16.7
	Type 2	7/24	29.2
	Type 3	8/24	33.3
	Type 4	4/24	16.7
	Total	24/24	100
Pain	24 h	20/24	83.3
	1 week	6/24	25.0
	3 months	0/24	0
Edema	24 h	22/24	91.7
	1 week	8/24	33.3
	3 months	0/24	0
Clinically stable zygomatic implants	24 h	48/48	100
	1 week	48/48	100
	3 months	48/48	100
Participants with serious complications	24 h	0/24	0
	1 week	0/24	0
	3 months	0/24	0

Postoperative clinical follow-up showed a progressive reduction in pain and edema. Pain was reported by 20 participants (83.3%) at 24 h and by 6 participants (25.0%) at 1 week; no participants reported pain at the 3-month assessment. Edema was observed in 22 participants (91.7%) at 24 h and in 8 participants (33.3%) at 1 week, with complete resolution by 3 months. All 48 zygomatic implants remained clinically stable at 24 h, 1 week, and 3 months. No serious complications were recorded at any follow-up visit (Table 2).

The reliability analysis demonstrated excellent intraobserver and interobserver agreement for all evaluated measurements. For the virtual coronal position, intraobserver ICC values ranged from 0.993 to 0.997, while interobserver ICC values were 0.996 at baseline and 0.995 at the two-week repeat assessment. For the actual coronal position, intraobserver ICC values ranged from 0.932 to 0.961, with interobserver ICC values of 0.958 and 0.919 at baseline and repeat assessment, respectively. For the virtual apical position, intraobserver ICC values ranged from 0.998 to 0.999, and interobserver ICC values were 0.999 and 0.998. For the actual apical position, intraobserver ICC values ranged from 0.993 to 0.998, whereas interobserver ICC values were 0.996 at both assessments. These findings confirmed the high repeatability and reproducibility of the radiographic measurements (Table 3).

**Table 3:** Interobserver and intraobserver reliability

Measurement	Position	Observer	Baseline (mm)	Two-week repeat (mm)	Intraobserver ICC (95% CI)
Coronal position	Virtual Plan (mm)	Observer (1)	0.891 ± 0.340	0.889 ± 0.342	0.997* (0.995–0.999)
		Observer (2)	0.892 ± 0.342	0.884 ± 0.346	0.995* (0.990–0.997)
		Observer (3)	0.896 ± 0.338	0.892 ± 0.344	0.993* (0.988–0.996)
		Interobserver ICC	0.996* (0.994–0.998)	0.995* (0.992–0.997)	
	Actual Position (mm)	Observer (1)	2.810 ± 0.126	2.809 ± 0.119	0.961* (0.932–0.978)
		Observer (2)	2.812 ± 0.127	2.809 ± 0.127	0.932* (0.883–0.962)
		Observer (3)	2.809 ± 0.133	2.807 ± 0.137	0.938* (0.891–0.965)
		Interobserver ICC	0.958* (0.933–0.975)	0.919* (0.874–0.951)	
Apical position	Virtual Plan (mm)	Observer (1)	0.785 ± 0.630	0.788 ± 0.635	0.999* (0.998–0.999)
		Observer (2)	0.786 ± 0.630	0.789 ± 0.624	0.998* (0.997–0.999)
		Observer (3)	0.780 ± 0.628	0.785 ± 0.634	0.998* (0.996–0.999)
		Interobserver ICC	0.999* (0.998–0.999)	0.998* (0.998–0.999)	
	Actual Position (mm)	Observer (1)	2.515 ± 0.470	2.517 ± 0.468	0.998* (0.996–0.999)
		Observer (2)	2.512 ± 0.460	2.511 ± 0.474	0.994* (0.990–0.997)
		Observer (3)	2.524 ± 0.466	2.517 ± 0.463	0.993* (0.988–0.996)
		Interobserver ICC	0.996* (0.993–0.998)	0.996* (0.993–0.997)	

*ICC* Intraclass correlation coefficient

^*^ICC > 0.90 indicates excellent reliability

3D superimposition of the preoperative virtual plans and postoperative CBCT scans demonstrated measurable deviations between the planned and actual implant positions. For the coronal position, the mean deviation was 1.989 ± 0.540 mm on the right side and 1.846 ± 0.160 mm on the left side. The overall mean coronal deviation was 1.917 ± 0.401 mm, with a 95% confidence interval of 1.801–2.034 mm, a median of 1.743 mm, an interquartile range of 0.397 mm, and an overall range of 1.457–2.783 mm.

For the apical position, the mean deviation was 1.757 ± 0.172 mm on the right side and 1.709 ± 0.588 mm on the left side. The overall mean apical deviation was 1.733 ± 0.429 mm, with a 95% confidence interval of 1.609–1.858 mm, a median of 1.763 mm, an interquartile range of 0.226 mm, and an overall range of 0.447–2.680 mm (Table 4).

**Table 4:** Coronal and apical accuracy of virtually planned versus actual zygomatic implant positions

Measurement	Side	Virtual Plan (mm)	Actual Position (mm)	Deviation (mm)	95% CI	Median	IQ	Range
Coronal position	Right	0.874 ± 0.483	2.863 ± 0.074	1.989 ± 0.540	1.761–2.217	1.697	1.134	1.457–2.783
	Left	0.911 ± 0.045	2.757 ± 0.147	1.846 ± 0.160	1.778–1.913	1.75	0.269	1.683–2.113
	Overall	0.893 ± 0.340	2.810 ± 0.127	1.917 ± 0.401	1.801–2.034	1.743	0.397	1.457–2.783
Apical position	Right	0.319 ± 0.100	2.076 ± 0.171	1.757 ± 0.172	1.684–1.829	1.758	0.186	1.437–2.083
	Left	1.248 ± 0.590	2.958 ± 0.076	1.709 ± 0.588	1.461–1.958	1.785	0.335	0.447–2.680
	Overall	0.784 ± 0.629	2.517 ± 0.465	1.733 ± 0.429	1.609–1.858	1.763	0.226	0.447–2.680

The mean virtual and actual positions also differed between the two sides. For the coronal position, the mean virtual values were 0.874 ± 0.483 mm on the right and 0.911 ± 0.045 mm on the left, whereas the corresponding actual values were 2.863 ± 0.074 mm and 2.757 ± 0.147 mm, respectively. For the apical position, the mean virtual values were 0.319 ± 0.100 mm on the right and 1.248 ± 0.590 mm on the left, compared with actual values of 2.076 ± 0.171 mm and 2.958 ± 0.076 mm, respectively.

## Discussion

The present study evaluated the accuracy of transferring virtually planned zygomatic implant positions into clinical reality using a static computer-aided design and manufacturing protocol. Our findings demonstrated that while all 48 zygomatic implants were successfully placed and remained clinically stable throughout 3 months follow-up period, statistically significant deviations were observed between the planned and actual implant positions. The overall mean coronal deviation was 1.917 ± 0.401 mm and the mean apical deviation was 1.733 ± 0.429 mm, with a consistent systematic shift of approximately 2.0 mm in the coronal plane across both sides. These results suggest that although virtual planning provides a valuable three-dimensional roadmap for zygomatic implant placement, complete translational accuracy remains challenging to achieve in this anatomically complex region, so the null hypothesis was rejected.

When contextualized within the existing literature, the accuracy reported in the present study appears consistent with, though slightly higher than, deviations documented in investigations employing comparable static guidance systems. Tahmaseb et al. [28], in their systematic review and meta-analysis of static computer-aided implant surgery, reported mean coronal deviations typically ranging between 1.0 and 1.5 mm for conventional implants, acknowledging that zygomatic implants represent a more technically demanding application due to their extended length and oblique trajectory. Similarly, Wang et al. [25], in their systematic scoping review of computer-assisted zygomatic implant surgery, highlighted that guided approaches generally improve accuracy over freehand placement, yet substantial variability persists depending on guide design, fixation stability, and surgical experience. González-Rueda et al. [30] compared static navigation, dynamic navigation, and mixed reality appliances for zygomatic implant placement in vitro, concluding that while all digital modalities enhanced positional accuracy, none eliminated deviation entirely. The slightly greater deviations observed in our clinical cohort compared with in vitro or conventional implant studies may be attributed to the cumulative effects of soft tissue thickness, guide stabilization challenges over the atrophic maxillary ridge, manual drilling sequence execution, and the inherent mobility of soft tissues under general anesthesia. Notably, Zingari et al. [31] demonstrated that ZAGA classification significantly influences the accuracy of digitally planned zygomatic implants, suggesting that variations in alveolar atrophy patterns and zygomatic anatomy may predispose to specific directional errors. Ghallab et al. [33] further emphasized that completely guided protocols tend to yield superior accuracy compared with partially guided approaches, underscoring the importance of comprehensive guidance throughout the entire osteotomy preparation.

The clinical significance of the approximately 2.0 mm coronal and 1.7 mm apical deviations warrant careful consideration. In zygomatic implant surgery, the implant trajectory must negotiate critical anatomical structures including the infraorbital neurovascular bundle, the maxillary sinus, and the orbital floor [20, 21]. While deviations of this magnitude did not result in any neurosensory disturbances, oroantral communications, or orbital complications in our cohort, they may carry implications for prosthetic rehabilitation and long-term biomechanical performance. Bedrossian et al. [17] clarified that zygomatic implants function through engagement of multiple cortical plates, and subtle alterations in entry point or apex position may modify stress distribution patterns along the implant body. Furthermore, Menchini-Fabris et al. [36], in their retrospective case series assessing template-guided zygomatic implant placement via cone-beam computed tomography, reported comparable deviations and emphasized that such errors, though statistically significant, often remain within clinically acceptable thresholds provided they do not compromise vital structures or prosthetic emergence profiles. Xing Gao et al. [35] similarly noted that digital planning accuracy in zygomatic implants must be interpreted within the context of the substantial safety margins afforded by the zygomatic bone’s volume and the prosthetic compensation available through angled multi-unit abutments.

The absence of serious complications in our series aligns with the evolving consensus regarding the safety of computer-assisted zygomatic implant surgery. Chrcanovic et al. [22] documented in their updated systematic review that zygomatic implants achieve high survival rates but remain susceptible to complications including sinusitis, paresthesia, and soft tissue dehiscence. Molinero-Mourelle et al. [20] specifically identified maxillary sinusitis as the most frequently reported complication, with incidence rates varying considerably across surgical techniques. In our study, no instances of sinusitis, implant mobility, or neuropathic complications were observed, supporting the contention that virtual planning and guided surgery may reduce the risk of iatrogenic injury by preoperatively mapping safe trajectories [25, 27]. Rocha et al. [23], in their meta-analysis comparing sinusitis rates following sinus lift versus zygomatic implant surgery, suggested that graftless zygomatic approaches may actually confer lower sinus-related morbidity when anatomical precision is optimized. The utilization of the buccal fat pad flap for implant shaft coverage, as described by Hernández-Alfaro et al. [32], further contributed to favorable soft tissue outcomes in selected cases, although this was not systematically required in our cohort.

The rationale for employing zygomatic implants in this patient population is firmly established within the contemporary reconstructive paradigm. Severe maxillary atrophy, characterized by both vertical and horizontal bone dimensions insufficient for conventional implant placement, traditionally necessitated invasive augmentation procedures including onlay grafting or sinus floor elevation [7, 8]. However, such interventions introduce donor-site morbidity, particularly when extraoral harvest sites such as the iliac crest are utilized [9] and prolong treatment duration considerably. Nedir et al. [10] described the transition toward graftless solutions as a paradigm shift in atrophic maxilla management, a concept subsequently validated by the ITI consensus report which endorsed zygomatic implants as a predictable alternative for patients with residual bone height ≤ 4 mm [15]. Long-term systematic reviews and meta-analyses have confirmed survival rates approaching 97% [16], attributing this success to the biomechanical engagement of four distinct cortical plates along the implant trajectory [17]. The present study contributes to this evidence base by demonstrating that virtual planning facilitates prosthetically driven placement while maintaining the graftless advantages inherent to zygomatic implant therapy [14].

The integration of CBCT virtual planning into zygomatic implant surgery represents a meaningful advancement toward precision and predictability. Fokas et al. [26] confirmed the reliability of linear measurements derived from CBCT for presurgical implant planning, establishing the foundational accuracy upon which three-dimensional planning software operates. D’haese et al. [27] reviewed the state of computer-guided implant surgery, highlighting its capacity to reduce operative time and minimize postoperative morbidity through preoperative visualization of anatomical constraints. In the specific context of zygomatic implants, Wu et al. [24] evaluated dynamic navigation systems and reported high reliability for complex trajectories, while Abouzeid et al. [29] proposed modified protocols merging published techniques to enhance guidance fidelity. Rinaldi and Ganz [34] introduced novel surgical guide designs specifically tailored to zygomatic anatomy, illustrating the rapid evolution of static guidance methodologies. Despite these technological advances, our findings suggest that a systematic coronal shift persists, potentially originating from guide seating discrepancies, tissue displacement during osteotomy, or the manual insertion torque applied to these lengthy implants. Han et al. [39] investigated scan body-assisted surface-based registration and identified residual errors in implant positioning even with advanced registration algorithms, implying that technological refinement must be accompanied by meticulous surgical execution.

The methodological strength of the present study was the standardization of both the virtual planning and surgical procedures. All procedures were performed by a single experienced implant surgeon with more than 10 years of experience in guided implant surgery. The same surgeon also conducted the virtual implant planning using the designated planning software. This approach minimized operator-dependent variability and enhanced the reliability and reproducibility of the measurements. However, because the procedures were performed by one highly experienced surgeon, the findings may not be directly generalizable to clinicians with different levels of training or experience [25, 28].

Several limitations of this study should be acknowledged. The single-arm accuracy design, wherein each participant served as their own control, precluded comparison with a freehand surgical cohort and limits the ability to quantify the relative advantage of virtual planning over conventional techniques. The sample size, though adequately powered for the primary accuracy outcome, may have been insufficient to detect rare complications or to perform robust subgroup analyses based on ZAGA classification, which Zingari et al. [31] identified as a significant modifier of placement accuracy. Additionally, measurements were performed at 2 weeks postoperatively, precluding assessment of whether initial deviations influenced long-term osseointegration, crestal bone levels, or prosthetic outcomes. Di Cosola et al. [37], in their long-term retrospective analysis, emphasized that clinical and radiologic data over extended follow-up periods are essential to validate the functional consequences of minor positional deviations. Thus, the study findings represent short-term results on positional accuracy and clinical status. Recent 3- to 5-year clinical and radiologic data on immediately loaded full-arch restorations supported by conventional implants combined with unilateral or bilateral zygomatic implants reported no zygomatic implant failures in either configuration, while prosthetic and rhinosinusitis-related events highlighted the need for multidimensional long-term follow-up [19].

A further limitation concerns uncertainty introduced during landmark identification and CBCT-to-plan superimposition. Intra- and interobserver reliability of landmark identification and the registration procedure were not separately quantified. Reliability estimates for repeated positional measurements do not establish the reproducibility of the registration step itself. Consequently, the observed coronal and apical deviations may reflect both surgical placement discrepancies and errors introduced during landmark identification, image registration, and measurement; the contribution of each source of error cannot be distinguished. The entire observed deviation therefore cannot be attributed to surgical placement alone. Future studies should independently repeat the complete landmark-identification and registration workflow and quantify its reliability and registration error.

## Conclusions

Within the limitations of this single-arm study, virtual surgical planning enabled the clinical transfer of planned zygomatic implant positions. No serious complications were observed during the three-month follow-up period; however, the sample size and follow-up duration were insufficient to establish treatment safety, efficacy, or long-term implant stability. These findings should therefore be interpreted as evidence of short-term positional accuracy only.

## Data Availability

The datasets used and/or analysed during the current study are available from the corresponding author on reasonable request.

## Abbreviations

CBCT: Cone-beam computed tomography
CP: Coronal plane
FHP: Frankfort horizontal plane
ITI: International Team for Implantology
REC: Research Ethics Committee
SD: Standard deviation
WHO: World Health Organization
ZAGA: Zygoma Anatomy-Guided Approach
3D: Three-dimensional

## References

[1] Gallucci GO, Hamilton A, Akhondi S, Pala K, Peña-Cardelles JF. Current State of Evidence for Implant Placement and Loading in Partially Edentulous Patients: A Systematic Review. Clin Implant Dent Relat Res. 2026;28:e70120. 10.1111/cid.70120 PMC12828728 41574557

[2] French D, Ofec R, Levin L. Long term clinical performance of 10 871 dental implants with up to 22 years of follow-up: A cohort study in 4247 patients. Clin Implant Dent Relat Res. 2021;23:289–297. 10.1111/cid.12994 PMC8359846 33768695

[3] Vaira LA, Biglio A, Roy M, Salzano G, Troise S, Abbate V, Mayo-Yanez M, Lechien JR, Piombino P, De Riu G. Full-arch rehabilitation of severely atrophic maxilla with additively manufactured custom-made subperiosteal implants: A multicenter retrospective study. J Craniomaxillofac Surg. 2024;52:991–998. 10.1016/j.jcms.2024.06.016 38871617

[4] Udeabor SE, Heselich A, Al-Maawi S, Alqahtani AF, Sader R, Ghanaati S. Current Knowledge on the Healing of the Extraction Socket: A Narrative Review. Bioengineering (Basel). 2023;10:1145. 10.3390/bioengineering10101145 PMC10604628 37892875

[5] Tallgren A. The continuing reduction of the residual alveolar ridges in complete denture wearers: a mixed-longitudinal study covering 25 years. 1972. J Prosthet Dent. 2003;89:427 − 35. 10.1016/s0022-3913(03)00158-6 12806317

[6] Whyte A, Boeddinghaus R. The maxillary sinus: physiology, development and imaging anatomy. Dentomaxillofac Radiol. 2019;48:20190205. 10.1259/dmfr.20190205 PMC6951102 31386556

[7] Urban I, Montero E, Sanz-Sánchez I, Palombo D, Monje A, Tommasato G, Chiapasco M. Minimal invasiveness in vertical ridge augmentation. Periodontol 2000. 2023;91:126–144. 10.1111/prd.12479 36700299

[8] Alshamrani AM, Mubarki M, Alsager AS, Alsharif HK, AlHumaidan SA, Al-Omar A. Maxillary Sinus Lift Procedures: An Overview of Current Techniques, Presurgical Evaluation, and Complications. Cureus. 2023;15:e49553. 10.7759/cureus.49553 PMC10753870 38156177

[9] Sudhakar KNV, Mohanty R, Singh V. Evaluation of Donor Site Morbidity Associated with Iliac Crest Bone Harvest in Oral and Maxillofacial, Reconstructive Surgery. J Clin Diagn Res. 2017;11:ZC28-ZC33. 10.7860/JCDR/2017/28688.10053 PMC5535479 28764289

[10] Nedir R, Nurdin N, Khoury P, El Hage M, Abi Najm S, Bischof M. Paradigm shift in the management of the atrophic posterior maxilla. Case Rep Dent. 2014;2014:486949. 10.1155/2014/486949 PMC4241701 25431689

[11] Luigi AE, Alessandro P, Enrico G. Zygoma Bone Shell Technique: A Proof-of-Concept Surgical Protocol in Human Cadaver for Bone Reconstruction After Zygomatic Implant Failure. Clin Exp Dent Res. 2025;11:e70093. 10.1002/cre2.70093 PMC11910187 40088480

[12] Gaur V, Doshi AG, Palka L. Zygomatic approach with single-piece implants: A technical note. Natl J Maxillofac Surg. 2022;13:153–161. 10.4103/njms.NJMS_274_20 PMC9326188 35911801

[13] Polido WD, Machado-Fernandez A, Lin WS, Aghaloo T. Indications for zygomatic implants: a systematic review. Int J Implant Dent. 2023;9:17. 10.1186/s40729-023-00480-4 PMC10313639 37391575

[14] Bedrossian E, Rangert B, Stumpel L, Indresano T. Immediate function with the zygomatic implant: a graftless solution for the patient with mild to advanced atrophy of the maxilla. Int J Oral Maxillofac Implants. 2006;21:937–942. 17190304

[15] Al-Nawas B, Aghaloo T, Aparicio C, Bedrossian E, Brecht L, Brennand-Roper M et al. ITI consensus report on zygomatic implants: indications, evaluation of surgical techniques and long-term treatment outcomes. Int J Implant Dent. 2023;9:28. 10.1186/s40729-023-00489-9 PMC10497463 37698775

[16] Brennand Roper M, Vissink A, Dudding T, Pollard A, Gareb B, Malevez C, Balshi T, Brecht L, Kumar V, Wu Y, Jung R. Long-term treatment outcomes with zygomatic implants: a systematic review and meta-analysis. Int J Implant Dent. 2023;9:21. 10.1186/s40729-023-00479-x PMC10322814 37405545

[17] Bedrossian E, Brunski J, Al-Nawas B, Kämmerer PW. Zygoma implant under function: biomechanical principles clarified. Int J Implant Dent. 2023;9:15. 10.1186/s40729-023-00483-1 PMC10287889 37347335

[18] Sales PH, Gomes MV, Oliveira-Neto OB, de Lima FJ, Leão JC. Quality assessment of systematic reviews regarding the effectiveness of zygomatic implants: an overview of systematic reviews. Med Oral Patol Oral Cir Bucal. 2020;25:e541-e548. 10.4317/medoral.23569 PMC7338062 32520920

[19] Menchini-Fabris GB, Toti P, Tommaso G, Paoleschi C, Paoleschi L, Covani U. Immediate Full-Arch Restorations Supported by Conventional Implants Plus Unilateral or Bilateral Zygomatic Implants: A 3- to 5-Year Retrospective Radiologic and Clinical Comparison. Int J Periodontics Restorative Dent. 2026;46:377–391. 10.11607/prd.7114 39808533

[20] Molinero-Mourelle P, Baca-Gonzalez L, Gao B, Saez-Alcaide LM, Helm A, Lopez-Quiles J. Surgical complications in zygomatic implants: A systematic review. Med Oral Patol Oral Cir Bucal. 2016;21:e751-e757. 10.4317/medoral.21357 PMC5116118 27694789

[21] Fukumura T, Takabayashi K, Akiyama K, Samukawa Y, Hoshikawa H. Detailed evaluation of the risk of infraorbital nerve injury in postoperative maxillary cyst surgery. Auris Nasus Larynx. 2025;52:229–233. 10.1016/j.anl.2025.03.005 40154119

[22] Chrcanovic BR, Albrektsson T, Wennerberg A. Survival and Complications of Zygomatic Implants: An Updated Systematic Review. J Oral Maxillofac Surg. 2016;74:1949-64. 10.1016/j.joms.2016.06.166 27422530

[23] Rocha RS, Vianna CP, Trojan LC, Padovan LEM, Dos Santos MCGL. Comparison of sinusitis rate after sinus lift procedure and zygomatic implant surgery: a meta-analysis. Oral Maxillofac Surg. 2024;28:63–77. 10.1007/s10006-023-01159-1 37266797

[24] Wu Y, Tao B, Lan K, Shen Y, Huang W, Wang F. Reliability and accuracy of dynamic navigation for zygomatic implant placement. Clin Oral Implants Res. 2022;33:362–376. 10.1111/clr.13897 PMC9305866 35113463

[25] Wang W, Yu X, Wang F, Wu Y. Clinical efficacy of computer-assisted zygomatic implant surgery: A systematic scoping review. J Prosthet Dent. 2025;134:1062–1074. 10.1016/j.prosdent.2023.10.032 38007293

[26] Fokas G, Vaughn VM, Scarfe WC, Bornstein MM. Accuracy of linear measurements on CBCT images related to presurgical implant treatment planning: A systematic review. Clin Oral Implants Res. 2018;29:393–415. 10.1111/clr.13142 30328204

[27] D’haese J, Ackhurst J, Wismeijer D, De Bruyn H, Tahmaseb A. Current state of the art of computer-guided implant surgery. Periodontol 2000. 2017;73:121–133. 10.1111/prd.12175 28000275

[28] Tahmaseb A, Wu V, Wismeijer D, Coucke W, Evans C. The accuracy of static computer-aided implant surgery: A systematic review and meta-analysis. Clin Oral Implants Res. 2018;29:416–435. 10.1111/clr.13346 30328191

[29] Abouzeid OEM, Gaber RM, Maergy HA, Hany HE, Abdelmohsen KM, Elkassaby MA, Taha MM. A modified protocol merging two published techniques for computer guided zygomatic implants surgery: a technical note. BMC Oral Health. 2024;24:1273. 10.1186/s12903-024-05010-1 PMC11515538 39449100

[30] González-Rueda JR, Galparsoro-Catalán A, de Paz-Hermoso VM, Riad-Deglow E, Zubizarreta-Macho Á, Pato-Mourelo J, Hernández-Montero S, Montero-Martín J. Accuracy of zygomatic dental implant placement using computer-aided static and dynamic navigation systems compared with a mixed reality appliance. An in vitro study. J Clin Exp Dent. 2023;15:e1035-e1044. 10.4317/jced.61097 PMC10767737 38186921

[31] Zingari F, Gallo F, Finamore R, Barone S, Giudice A. Influence of ZAGA Classification on the Accuracy of 150 Digitally Planned Zygomatic Implants Placement. Clin Implant Dent Relat Res. 2026;28:e70117. 10.1111/cid.70117 41510633

[32] Hernández-Alfaro F, Ragucci GM, Valls-Ontańón A, Hamawandi AA, Bertos-Quílez J. Extramaxillary Zygomatic Implant Coverage with a Pedicled Buccal Fat Pad Flap Through a Tunnel Approach: A Prospective Case Series. Int J Oral Maxillofac Implants. 2022;37:400–406. 10.11607/jomi.9332 35476870

[33] Ghallab MK, Shabaan AA, Al-Mahalawy H, Taha M. Accuracy of Zygomatic Implant Placement Using Completely Versus Partially Guided. BMC Oral Health. 2026;26:826. 10.1186/s12903-026-08436-x PMC13169831 42087122

[34] Rinaldi M, Ganz SD. Computer-Guided Approach for Placement of Zygomatic Implants: Novel Protocol and Surgical Guide. Compend Contin Educ Dent. 2019;40:e1-e4. 30829495

[35] Xing Gao B, Iglesias-Velázquez O, G F Tresguerres F, Rodríguez González Cortes A, F Tresguerres I, Ortega Aranegui R, López-Pintor RM, López-Quiles J, Torres J. Accuracy of digital planning in zygomatic implants. Int J Implant Dent. 2021;7:65. 10.1186/s40729-021-00350-x PMC8295422 34291352

[36] Menchini-Fabris GB, Toti P, Park YM, Leusink F, Cosola S, Grandi T. Accuracy of template-guided zygomatic implant placement: a retrospective case series using cone-beam computed tomography. Oral Maxillofac Surg. 2026;30:108. 10.1007/s10006-026-01598-6 42420690

[37] Di Cosola M, Ballini A, Zhurakivska K, Ceccarello A, Nocini R, Malcangi A, Mori G, Lo Muzio L, Cantore S, Olivo A. Retrospective Analysis of Clinical and Radiologic Data Regarding Zygomatic Implant Rehabilitation with a Long-Term Follow-Up. Int J Environ Res Public Health. 2021;18:12963. 10.3390/ijerph182412963 PMC8701901 34948572

[38] González-Rueda JR, Galparsoro-Catalán A, de Paz-Hermoso VM, Riad-Deglow E, Zubizarreta-Macho Á, Pato-Mourelo J et al. Accuracy of zygomatic dental implant placement using computer-aided static and dynamic navigation systems compared with a mixed reality appliance. An in vitro study. J Clin Exp Dent. 2023;15:e1035-e1044. 10.4317/jced.61097 PMC10767737 38186921

[39] Han MD, Graca S, Ghunaim D, Ahmadian L. An in vitro accuracy study on scan body-assisted surface based registration for conventional and zygomatic dental implants. Sci Rep. 2025;15:43129. 10.1038/s41598-025-26936-8 PMC12678584 41339411

